# Supplementary material for: CDC20B is required for deuterosome-mediated centriole production in multiciliated cells
Source: Nat Commun. 2018 Nov 7;9:4668. doi: 10.1038/s41467-018-06768-z (PMC6220262; doi:10.1038/s41467-018-06768-z)
Supplement: Supplementary file 1 — Supplementary Information [file 41467_2018_6768_MOESM1_ESM.pdf]

Supplementary information

CDC20B is required for deuterosome-mediated centriole production in multiciliated cells

Revinski et al.

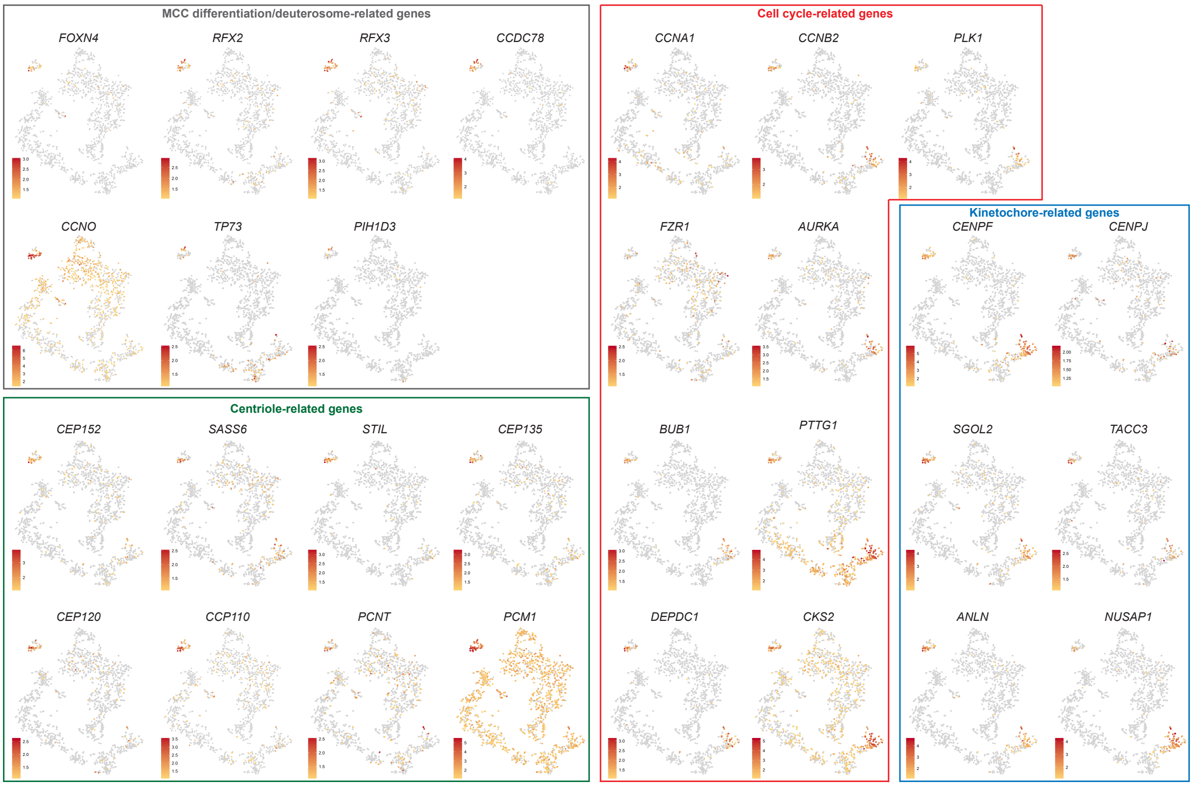

Supplementary Figure 1: Single cell RNA-seq analysis of HAECs.

tSNE plots for a selection of genes expressed at the single-cell level, in deuterosomal-stage differentiating HAECs. Genes were grouped into functional categories.

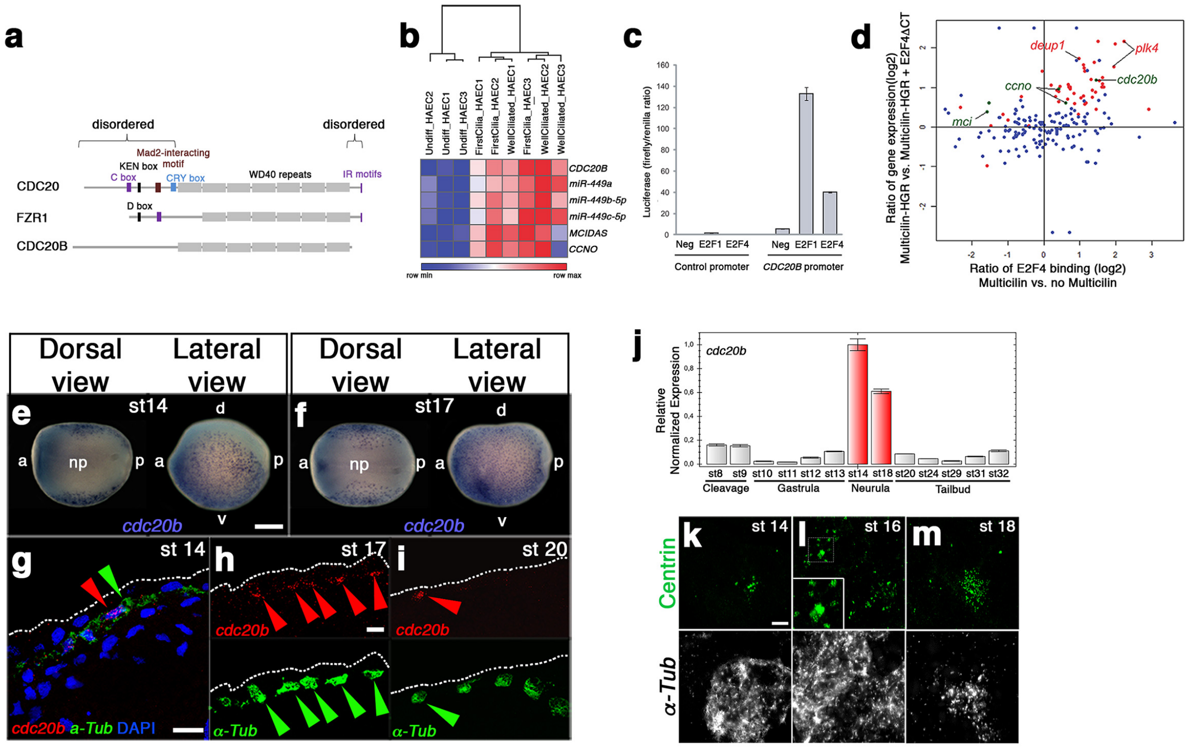

Supplementary Figure 2: Structure, regulation and spatio-temporal expression of *cdc20b*. **(a)** Domain composition of CDC20 family members. The C box and IR motifs in CDC20 and FZR1 serve as APC/C binding domains. The KEN box and the Cry box in CDC20, and the D box in FZR1 are involved in their regulation by degradation. The Mad2-interacting motif in CDC20 is important for its regulation by the spindle assembly checkpoint. WD40 repeats are involved in substrate recognition. Note that CDC20B lacks degradation motifs and the APC/C binding domains present in CDC20 and FZR1. (**b**) Heatmap of gene expression measured by RNA-seq or small RNA-seq on 3 independent HAEC differentiation time courses (HAEC1 to HAEC3). Normalized read counts were Log2-transformed and median-centered by gene. Hierarchical clustering (Euclidian distance) was performed on samples. The scale color bar indicates the minimum and maximum values per row. (**c**) Promoter luciferase reporter assay. Promoter-associated firefly luciferase was normalized to constitutive renilla luciferase. Control and *CDC20B* promoter were co-expressed with a plasmid expressing E2F1 or E2F4, or a negative control. Bars represent the average of 3 independent experiments. Error bars represent

the standard deviation. (**d**) Ratio of gene expression (Multicilin-HGR vs. Multicilin-HGR +E2F4ΔCT) vs. ratio of E2F4 binding (Multicilin vs. no Multicilin). E2F4ΔCT prevents the formation of transcriptionally active Multicilin/E2F complexes. Centriole-related genes are highlighted in red. Genes from the multiciliary locus are highlighted in green. The graph was built by mapping and quantifying previously published raw data[9]. (**e**,**f**) *cdc20b* whole-mount *in situ* hybridization in early *Xenopus laevis* neurula st14 and st17, respectively. *cdc20b* mRNA is expressed in epidermal cells but not in the neural plate (np), as revealed on dorsal views. a: anterior, p: posterior, d: dorsal, v: ventral. (**g**-**i**) *cdc20b* (red) and *α-Tubulin* (α-*Tub,* green) double fluorescent *in situ* hybridization (FISH) on sectioned embryos at st14 (**g**), st17 (**h**) and st20 (**i**). Red and green arrows point immature MCCs co-expressing *cdc20b* and *α-Tub*. Nuclei are revealed by DAPI staining in blue. White dotted lines indicate the surface of the epidermis. Note that the majority of MCCs become negative for *cdc20b* expression at st20. (**j**) RT-qPCR showing the relative expression of *cdc20b* from st8 (mid-blastula transition) until tadpole st32 normalized to *ODC* expression. Red bars indicate the peak of *cdc20b* transcript accumulation between st14 and st18, when centriole amplification occurs. (**k**-**m**) To reveal the dynamics of centriole multiplication, MCCs were stained by α-*Tub* FISH and by immunostaining against Centrin. Multiple Centrin-positive foci were detected at st14, marking the onset of centriologenesis. Procentriole aggregates, presumably organized around deuterosomes were clearly visualized at st16 (inset). Dispersed multiple centrioles were detected at st18. Scale bars: 250µm (**e**), 20µm (**g**,**h**), 5µm (**k**).

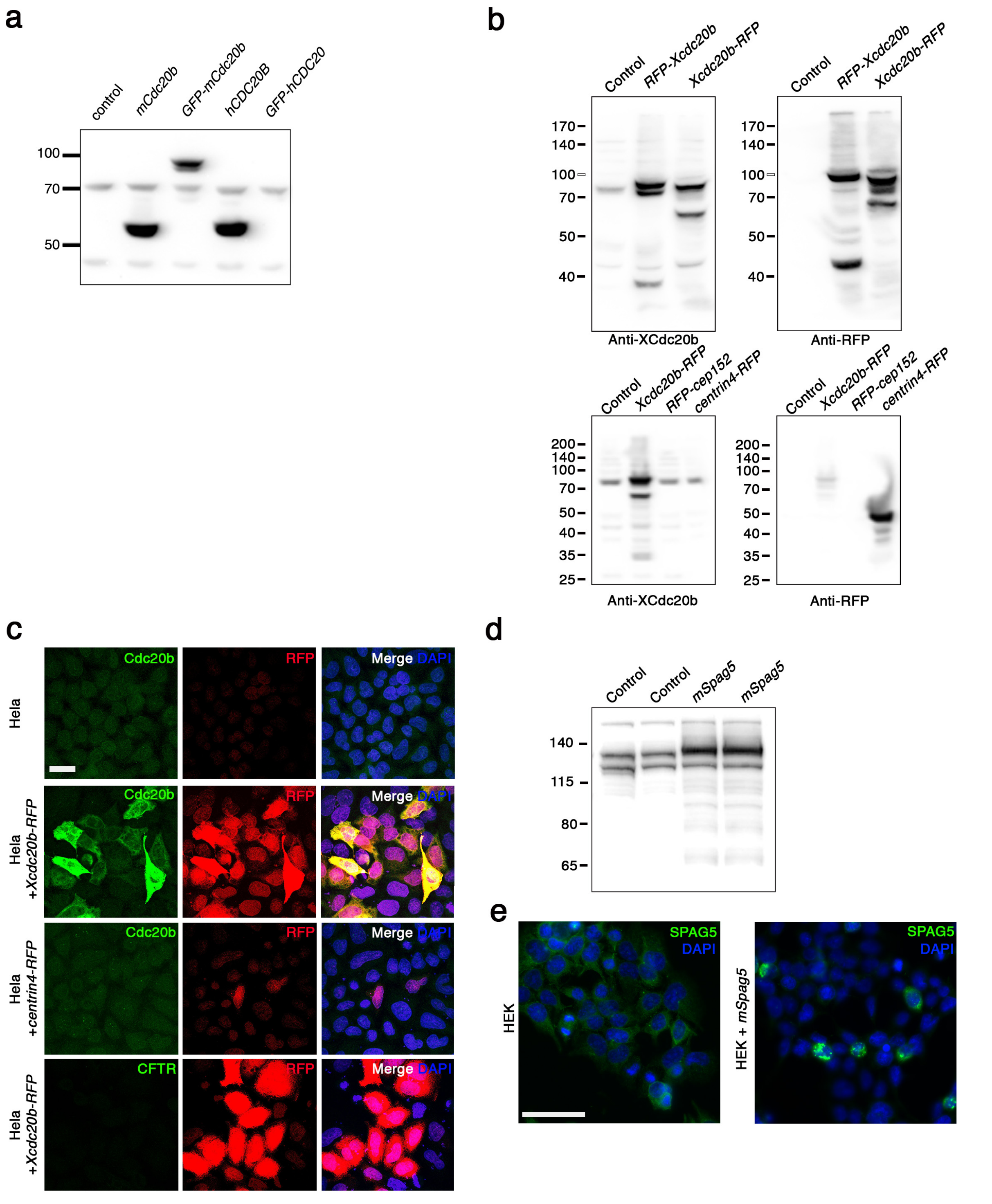

Supplementary Figure 3: Antibody validations

(a) COS1 cells were transfected with vectors coding for the indicated proteins and immunoblot was performed using Proteintech rabbit antibody raised against human CDC20B. This antibody recognized human and mouse CDC20B but did not cross-react with human CDC20. (**b**) Hela cells were transfected with vectors coding for the indicated proteins and immunoblot was

performed using a custom-made rabbit antibody raised against *Xenopus* CDC20B. (**c**) Hela cells were transfected with vectors coding for the indicated proteins and immunostainings were performed using the antibodies indicated on the photographs. Note that the antibody directed against *Xenopus* CDC20B did not cross-react with the centriole marker Centrin4. (**d**) HEK cells were transfected in duplicate with pCMV6-mSpag5, lysed 24 hours later and western blot was performed using proteintech rabbit polyclonal antibody raised against human SPAG5. (**e**) HEK cells were transfected with pCMV6-mSpag5, fixed with methanol 24 hours later and immunostained using proteintech rabbit polyclonal antibody raised against human SPAG5. This antibody cross-reacted with mouse SPAG5. Scale bars: 20µm (**c**), 50µm (**e**).

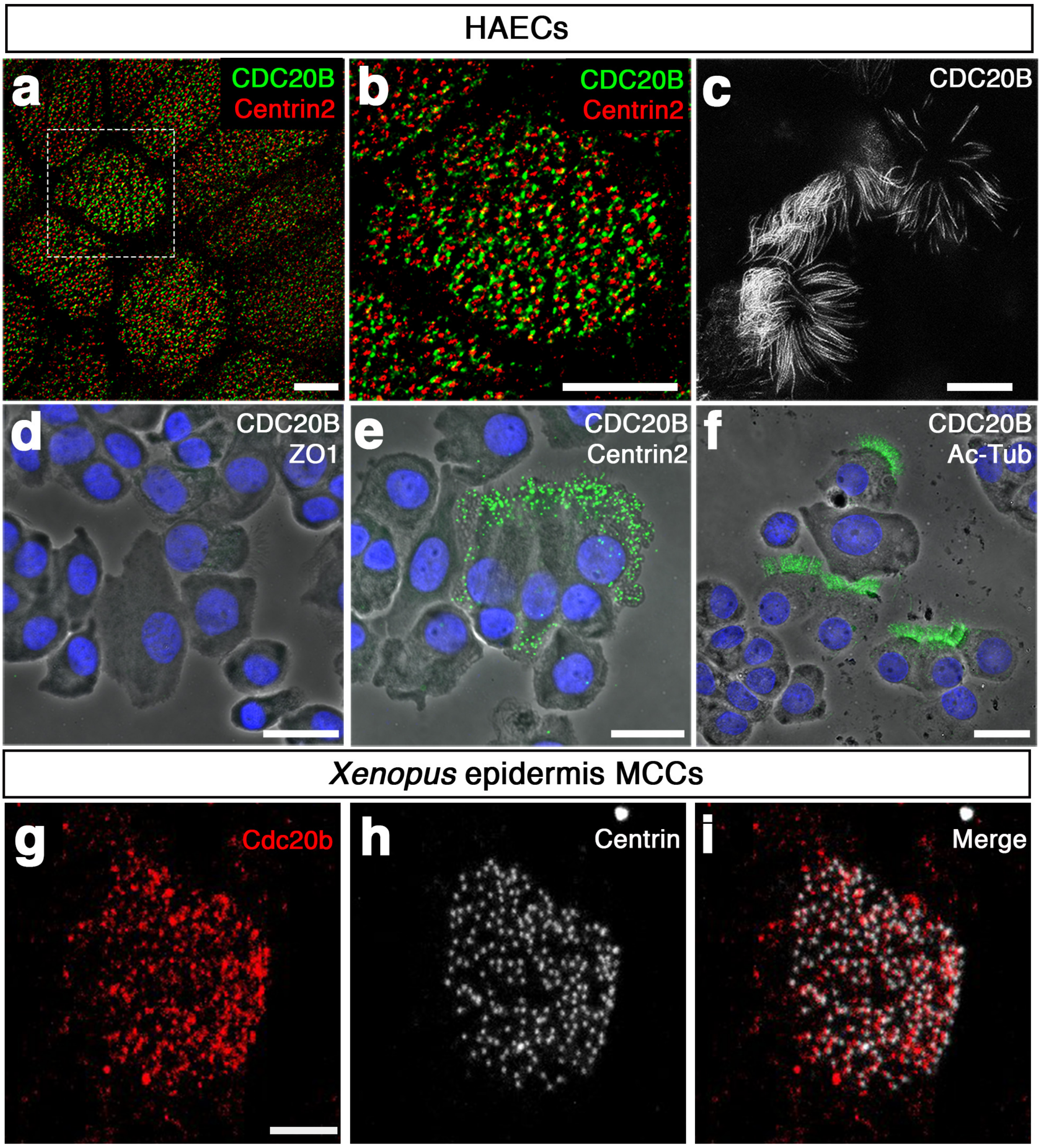

Supplementary Figure 4: CDC20B localization in mature MCCs.

(a-f) CDC20B sub-cellular localization in human mature MCCs. (**a**,**b**) ALI day 21 HAECs were fixed in methanol, and immunostained against CDC20B and Centrin2. STED super-resolution microscopy revealed the association of CDC20B to BBs. (**c**) ALI day 21 HAECs were fixed in paraformaldehyde, and immunostained against CDC20B. STED super-resolution microscopy revealed the association of CDC20B with cilia. (**d**-**f**) DuoLink Assays on fully

differentiated HAECs after cytospin. (**d**) Assay with CDC20B and ZO-1 antibodies was used as negative control. (**e**) Assay with CDC20B and Centrin2 (BBs) antibodies. (**f**) Assay with CDC20B and Acetylated-α-Tubulin (cilia) antibodies. Interaction between antibodies separated by less than 40nm generated green fluorescent signal. Nuclei are stained in blue. (**g**-**i**) **Cdc20b sub-cellular localization in *Xenopus* mature MCCs.** 4-cell *Xenopus* embryos were injected with *Multicilin-hGR* mRNA, induced with dexamethasone at stage 10.5 to activate Multicilin and immunostained for CDC20B (**g**) and Centrin (**h**) at stage 23. Scale bars: 5µm (**a**-**c**), 20µm (**d**-**f**), 5µm (**g**).

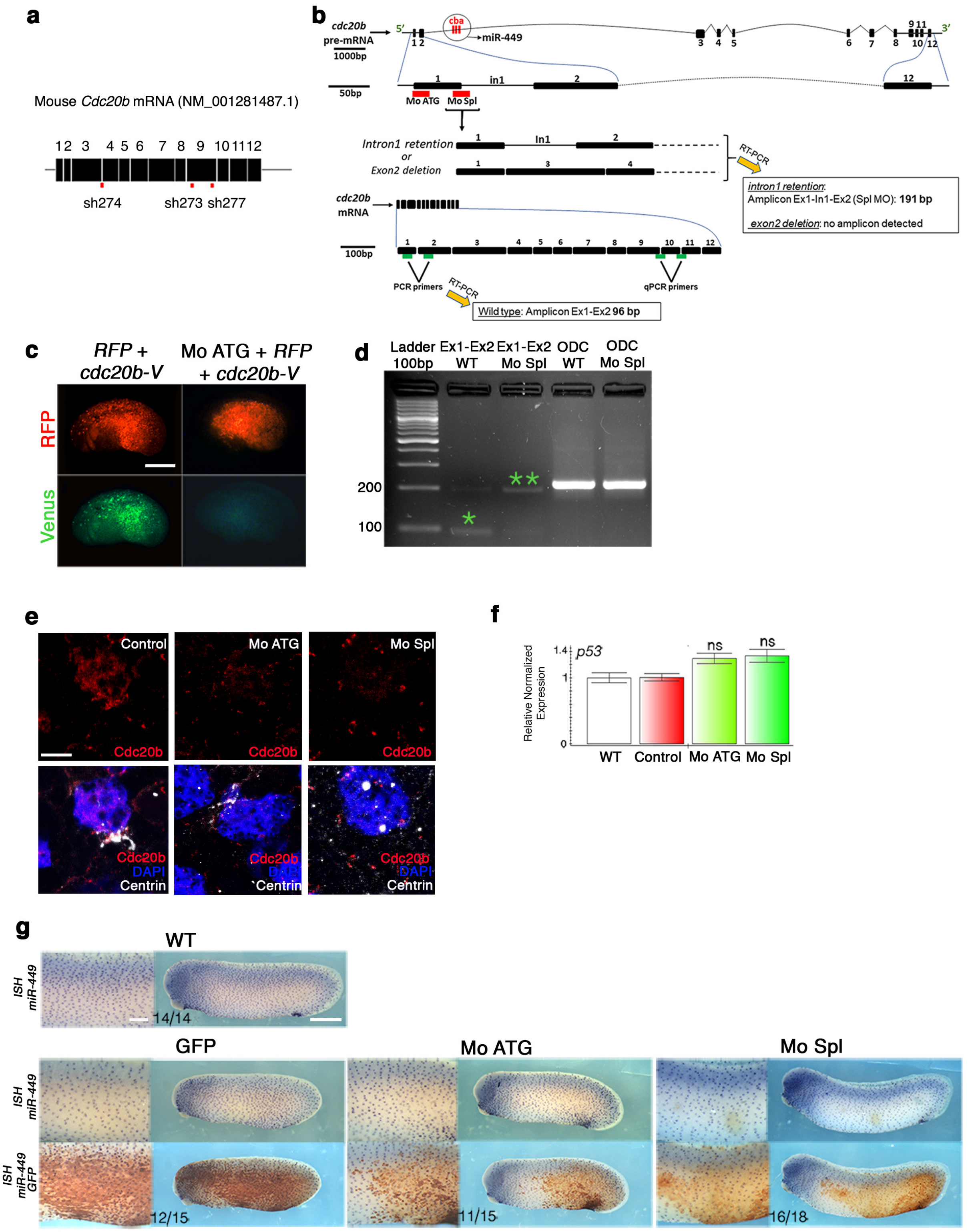

Supplementary Figure 5: *cdc20b* knockdown in mouse and *Xenopus*.

(**a**) Schematic representation of mouse *Cdc20b* mRNA and position of shRNAs used in this study. Note that sh274 targets the junction between exons 3 and 4, ruling out possible

interference with the production of miR-449 molecules from the *Cdc20b* pre-mRNA. (**b**) Schematic representation of *Xenopus cdc20b* pre-mRNA with introns, exons and miR-449abc relative position and size. Red horizontal bars below exon1 show the position of *cdc20b* Mo ATG and Mo Spl. On the bottom, green horizontal bars indicate RT-PCR and qPCR primer positions. (**c**) The efficiency of Mo ATG was verified through fluorescence extinction of co-injected *cdc20b-Venus*. (**d**) RT-PCR confirmed that Mo Spl caused intron1 retention (amplicon=191bp; double green stars), which is expected to introduce a premature stop codon and to produce a Cdc20b protein lacking 96% of its amino-acids, likely to undergo unfolded protein response-mediated degradation. (**e**) Immunostaining with the anti-*Xenopus* CDC20B antibody confirmed that both Mo ATG and Mo Spl severely down-regulated CDC20B protein expression in st18 MCCs. (**f**) RTqPCR revealed that neither *cdc20b* mopholinos caused significant *p53* transcript up-regulation, a non-specific response sometimes detected in zebrafish embryos subjected to morpholinos. Four independent experiments were carried out to check *p53* expression in morphant conditions. (**g**) miR-449 expression revealed by whole-mount *in situ* hybridization with LNA probes was not perturbed in the presence of either morpholinos. Embryos were photographed before (top) and after (bottom) staining against co-injected GFP-CAAX to be able to detect miR-449 staining. The number of embryos showing normal miR-449 expression over the total number of embryos analyzed is indicated on the photographs. Scale bars: 500µm (**c**), 5µm (**e**), 500µm (**g**, whole embryo), 80µm (**g**, zoom).

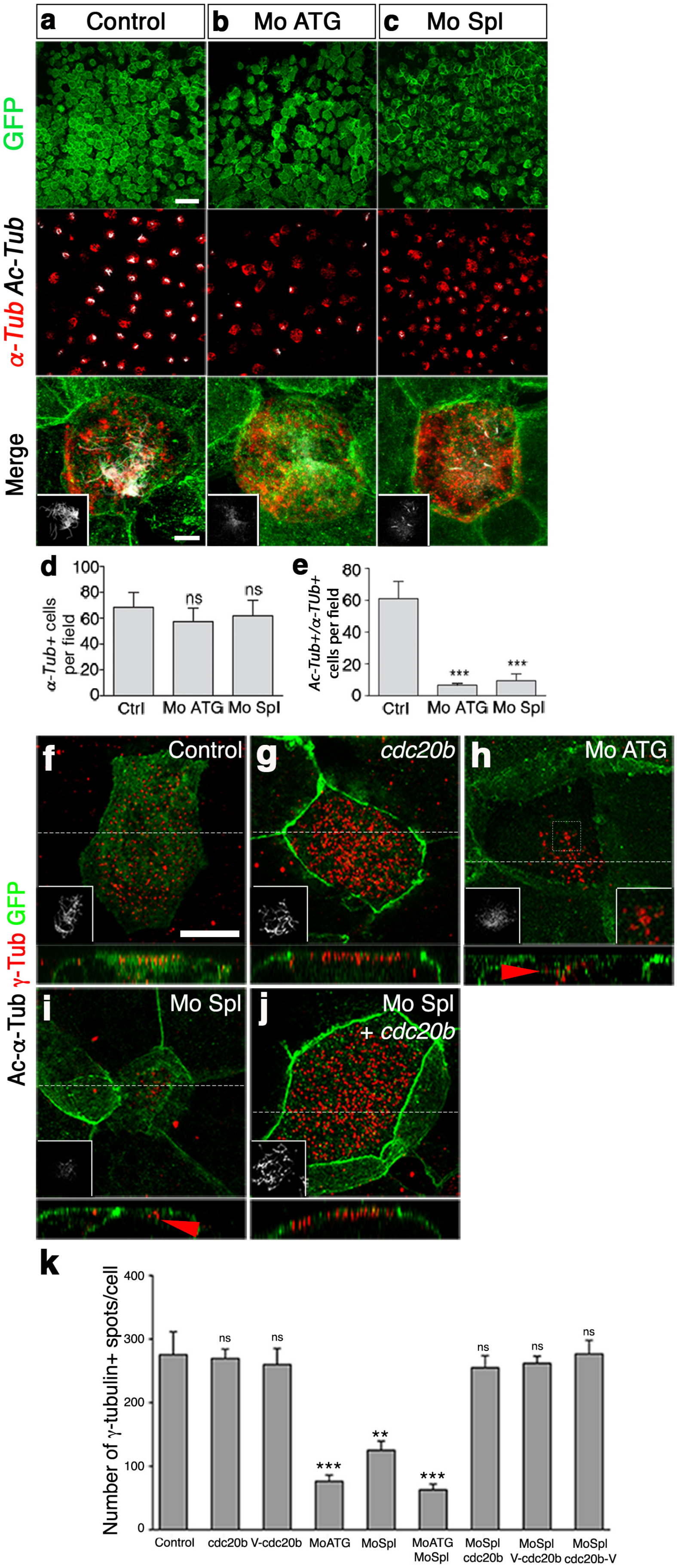

a Control
b Mo ATG
c Mo Spl
GFP
α-Tub Ac-Tub
Merge
d
α-Tub+ cells per field
100
80
60
40
20
0
ns
ns
Ctrl
Mo ATG
Mo Spl
e
Ac-Tub+/α-TUb+ cells per field
80
60
40
20
0
***
***
Ctrl
Mo ATG
Mo Spl
f Control
g cdc20b
h Mo ATG
i Mo Spl
j Mo Spl + cdc20b
Ac-α-Tub γ-Tub GFP
k
Number of γ-tubulin+ spots/cell
400
300
200
100
0
ns
ns
***
**
***
ns
ns
ns
Control
cdc20b
V-cdc20b
MoATG
MoSpl
MoATG MoSpl
MoSpl cdc20b
MoSpl V-cdc20b
MoSpl cdc20b-V

Supplementary Figure 6: *cdc20b* knockdown impairs multiciliogenesis in *Xenopus*.

(**a**-**e**) 8-cell embryos were injected in presumptive epidermis with *cdc20b* morpholinos and *GFP-CAAX* mRNA (injection tracer) as indicated. Control was provided by *GPF-CAAX* injection alone. Embryos at tailbud st25 were processed for fluorescent staining against GFP (green), Acetylated α-Tubulin (cilia, white) and *α-Tub* mRNA (MCC marker, red). Insets on merged panels show cilia staining. Note that *cdc20b* morphant MCCs maintain expression of fate marker *α-Tub* but poorly grow cilia. (**d**) Bar graph showing quantification of *α-Tub*/GFP double positive cells per field of observation. (**e**) Bar graph showing quantification of *α-Tub*/Ac-Tub/GFP triple positive cells per field of observation. 10 fields corresponding to 10 different embryos were analyzed for each condition. (**f**-**k**) 8-cell embryos were injected in presumptive epidermis with *cdc20b* morpholinos, *GFP-CAAX* and *cdc20b* mRNAs as indicated, and immunostained at tailbud st25 against GFP (injection tracer, green), γ-tubulin (BBs, red) and Acetylated α-Tubulin (cilia, white, left insets). Right inset in **h**: zoom on a stalled deuterosomal figure. z-projections made along white dotted lines are shown in bottom panels. Arrowheads point undocked BBs. (**k**) Bar graph showing the quantification of γ-tubulin spots per MCC. As two individual γ-tubulin spots are detected around each basal body, twice as many spots are usually counted as compared to Centrin (Fig. 5j-o). Note that BB numbers were restored to normal levels in *cdc20b* Spl morphants injected with tagged and untagged versions of *cdc20b*. Scale bars: 50µm (**a**, top), 5µm (**a**, bottom), 5µm (**f**).

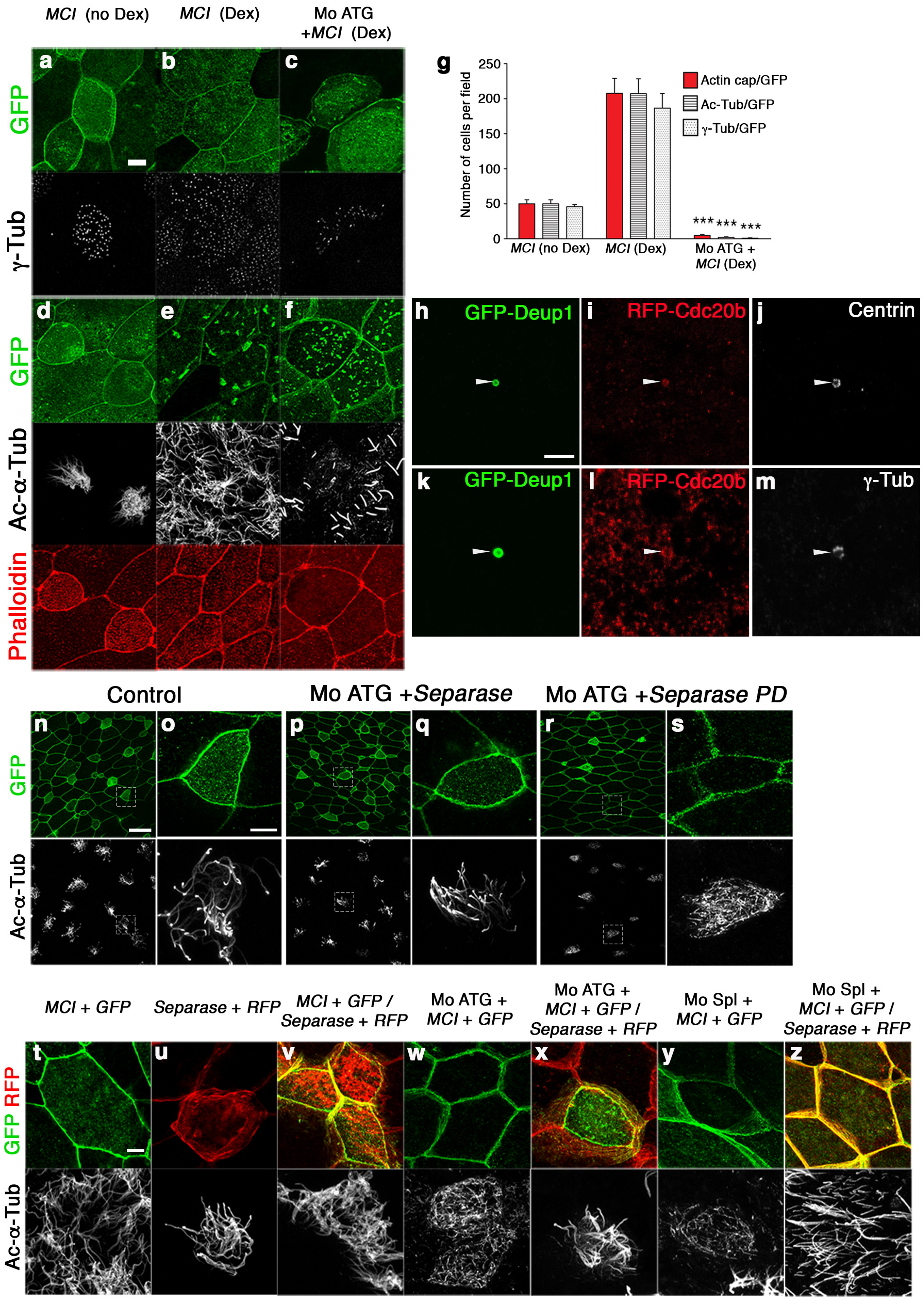

Supplementary Figure 7: *cdc20b* knockdown prevents multiciliogenesis induced by Multicilin, and is counteracted by Separase overexpression.

(**a-g**) ***cdc20b* knockdown prevents multiciliogenesis induced by Multicilin.** 8-cell embryos were injected in presumptive epidermis with *Multicilin-hGR* mRNA (*MCI*) and *cdc20b* Mo ATG, as indicated. *GFP-GPI* mRNA was co-injected as a tracer. MCI-hGR-injected embryos were induced with dexamethasone at st11. To check the efficiency of MCI induction some embryos were not treated with dexamethasone and served as controls (no DEX). Embryos were fixed at tailbud st25, and were stained against GFP (green) and γ-Tubulin (basal bodies, white)(**a-c**), or against GFP (green), phalloidin (apical actin, red), and Acetylated-α-Tubulin (cilia, white)(**d-f**). Note that *cdc20b* morphant MCI-induced MCCs failed to amplify centrioles, to maintain a proper actin cap, and to grow cilia. (**g**) Bar graph showing the quantification of GFP-positive cells that displayed normal actin, basal body and cilium staining. 5 fields (40x) per condition were analyzed. (**h-m**) **Deup1 recruits CDC20B in centriole amplification platforms.** 8-cell embryos were injected in presumptive epidermis with *Multicilin-hGR*, *RFP-CDC20B*, and *GFP-Deup1*mRNAs. Multicilin activity was induced with dexamethasone at st11, embryos were fixed at st18 and stained for GFP, RFP, Centrin (centrioles) or γ-Tubulin (deuterosome). White arrowheads point a centriole amplification platform positive for GFP-Deup1, which incorporates RFP-CDC20B, consistent with their capacity to form a complex (Fig. 6c). (**n-z**) **Wild-type but not protease-dead Separase rescues multiciliogenesis in MCCs deficient for Cdc20b.** (**n-s**) 8-cell embryos were injected in presumptive epidermis with *GFP-GPI* mRNA, human *Separase* mRNA, and *cdc20b* Mo ATG, as indicated. Immunofluorescence against GFP (injection tracer, green), and Acetylated-α-Tubulin (cilia, white) was performed at tailbud st25. Cells in dotted squares were blown up for better visualization. Note that multiciliogenesis was rescued in *cdc20b* morphant MCCs by wild-type (**p**,**q**) but not protease-dead Separase (**r**,**s**). (**t-z**) 4-cell embryos were injected in one ventral blastomere (presumptive epidermis) with *MCI-hGR* and *GFP-GPI* mRNAs, in the presence or not of *cdc20b* morpholinos, as indicated. Next, at 16-cell stage, half of those embryos were

injected with human *Separase* and RFP mRNAs in one ventral-animal blastomere. This setup was designed to avoid co-injection of *cdc20b* morpholinos with *Separase* mRNA, ruling out non-specific interference *in vitro* between these reagents. MCI-hGR-injected embryos were induced with dexamethasone at st11. All embryos were fixed at tailbud st25 and stained for GFP (*cdc20b* Mo tracer, green), RFP (*Separase* tracer, red) and Acetylated-α-Tubulin (cilia, white). Note that multiciliogenesis failed in MCI-induced *cdc20b* morphant MCCs (**w**,**y**). The presence of Separase rescued multiciliogenesis in MCI-induced *cdc20b* morphant MCCs (**x**,**z**). Scale bars: 5μm (**a**, **h**, **o**, **t**), 20μm (**n**).

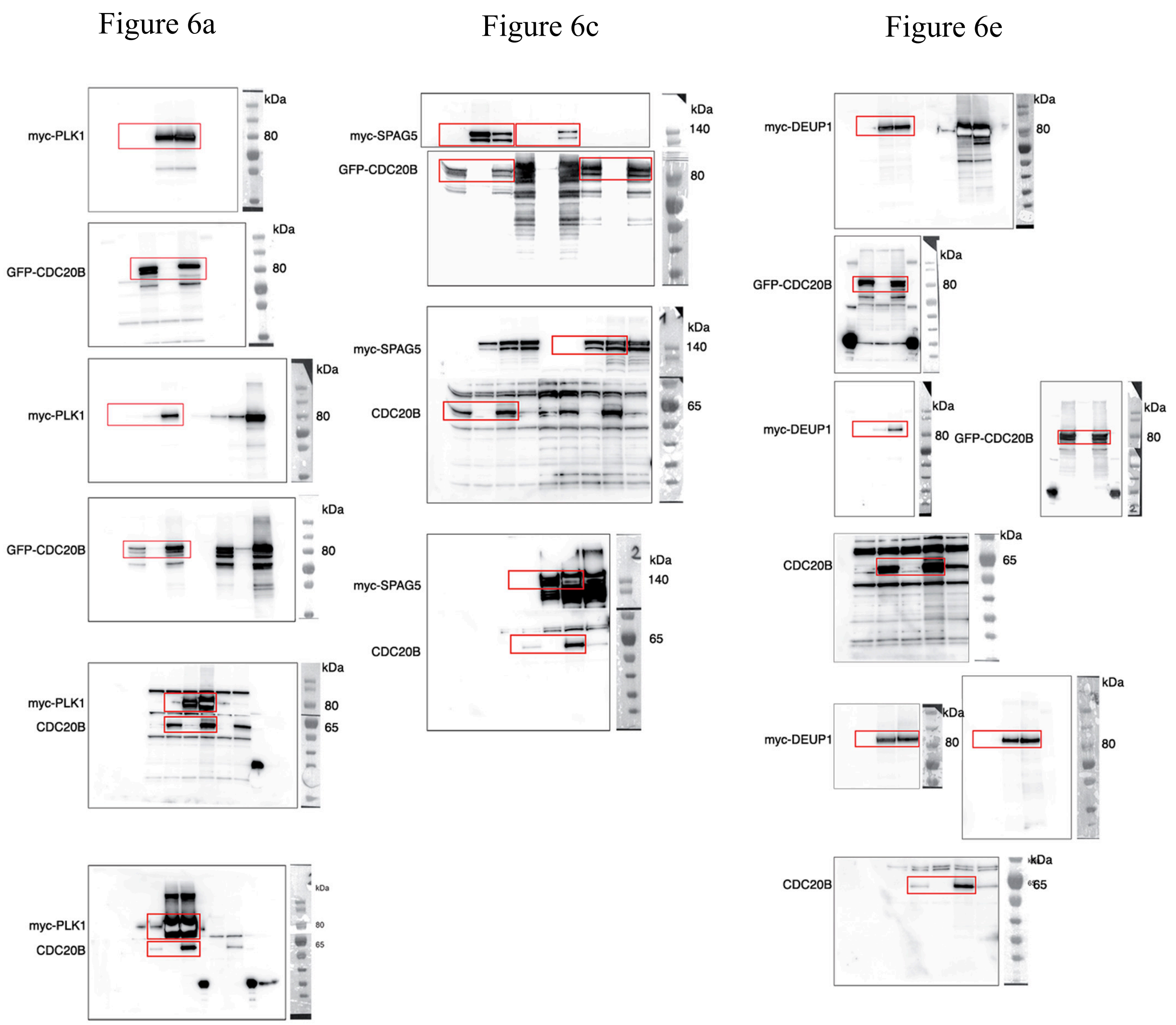

Supplementary Figure 8: Uncropped Western blots.

Red boxes mark the parts of the Western blot images that are shown in the indicated parts of Figure 6.

G1/S	
Symbol	Ensembl
ORC1	ENSG00000085840
ZNF367	ENSG00000165244
ADAMTS1	ENSG00000154734
CCNE2	ENSG00000175305
CDC25A	ENSG00000164045
RECQL4	ENSG00000160957
DTL	ENSG00000143476
CDC6	ENSG00000094804
CCNE1	ENSG00000105173
MCM2	ENSG00000073111
GINS3	ENSG00000181938
CHAF1B	ENSG00000159259
WDR76	ENSG00000092470
MCM6	ENSG00000076003
CLSPN	ENSG00000092853
CDCA7	ENSG00000144354
OSBPL6	ENSG00000079156
RAB23	ENSG00000112210
PLCXD1	ENSG00000182378
SKP2	ENSG00000145604
MDM1	ENSG00000111554
GINS2	ENSG00000131153
E2F1	ENSG00000101412
MCM5	ENSG00000100297
SNHG10	ENSG00000247092
HSF2	ENSG00000025156
UBR7	ENSG00000012963
RMI2	ENSG00000175643
NUP43	ENSG00000120253
ACD	ENSG00000102977
ZMYND19	ENSG00000165724
MSH2	ENSG00000095002
CDCA7L	ENSG00000164649
KIAA1586	ENSG00000168116
PMS1	ENSG00000064933
UNG	ENSG00000076248
KIAA1147	ENSG00000257093
POLD3	ENSG00000077514
ANKRD10	ENSG00000088448
CHAF1A	ENSG00000167670
BARD1	ENSG00000138376
INTS8	ENSG00000164941
APEX2	ENSG00000169188
ACYP1	ENSG00000119640
MRI1	ENSG00000037757
INSR	ENSG00000171105
TOPBP1	ENSG00000163781
FAM105B	ENSG00000154124
NPAT	ENSG00000149308
PCDH7	ENSG00000169851
GMNN	ENSG00000112312
RNPC3	ENSG00000185946
RNF113A	ENSG00000125352
FAM122A	ENSG00000187866
CAPN7	ENSG00000131375
TIPIN	ENSG00000075131
C14orf142	ENSG00000170270
LNPEP	ENSG00000113441
USP53	ENSG00000145390
PANK2	ENSG00000125779
VPS72	ENSG00000163159
DIS3	ENSG00000083520

S	
Symbol	Ensembl
ANKRD18A	ENSG00000273170
REEP1	ENSG00000068615
DEPDC7	ENSG00000121690
CDC7	ENSG00000097046
DNA2	ENSG00000138346
EXO1	ENSG00000174371
KDELC1	ENSG00000134901
ANKRD18A	ENSG00000180071
BRIP1	ENSG00000136492
PKMYT1	ENSG00000127564
CDC45	ENSG00000093009
C11orf82	ENSG00000165490
BLM	ENSG00000197299
RAD51	ENSG00000051180
CCDC150	ENSG00000144395
CDCA5	ENSG00000146670
CPNE8	ENSG00000139117
MCM8	ENSG00000125885
ESCO2	ENSG00000171320
GOLGA8B	ENSG00000215252
ASF1B	ENSG00000105011
FANCA	ENSG00000187741
INTS7	ENSG00000143493
POLA1	ENSG00000101868
FANCI	ENSG00000140525
RRM2	ENSG00000171848
TTLL7	ENSG00000137941
RAD51AP1	ENSG00000111247
KAT2B	ENSG00000114166
CHML	ENSG00000203668
BRCA1	ENSG00000012048
ABHD10	ENSG00000144827
TYMS	ENSG00000176890
PRIM1	ENSG00000198056
TTC31	ENSG00000115282
E2F8	ENSG00000129173
CENPQ	ENSG00000031691
PHTF1	ENSG00000116793
MASTL	ENSG00000120539
OSGIN2	ENSG00000164823
GOLGA8A	ENSG00000175265
PHTF2	ENSG00000006576
BBS2	ENSG00000125124
BMI1	ENSG00000168283
FEN1	ENSG00000168496
RMI1	ENSG00000178966
NSUN3	ENSG00000178694
KAT2A	ENSG00000108773
CENPM	ENSG00000100162
ZWINT	ENSG00000122952
ORC3	ENSG00000135336
KIAA1598	ENSG00000187164
BIVM	ENSG00000134897
DNAJB4	ENSG00000162616
CCDC84	ENSG00000186166
DCAF16	ENSG00000163257
NUP160	ENSG00000030066
RFC2	ENSG00000049541
CDKN2AIP	ENSG00000168564
UBE2T	ENSG00000077152
DHFR	ENSG00000228716
PTAR1	ENSG00000188647
RAD18	ENSG00000070950
OGT	ENSG00000147162
EZH2	ENSG00000106462
C5orf42	ENSG00000197603
LYRM7	ENSG00000186687
CCDC14	ENSG00000175455
NAB1	ENSG00000138386
SP1	ENSG00000185591
RPA2	ENSG00000117748
RBBP8	ENSG00000101773
RRM1	ENSG00000167325
FAM178A	ENSG00000119906
SAP30BP	ENSG00000161526
NT5DC1	ENSG00000178425
CERS6	ENSG00000172292
ZBED5	ENSG00000236287
MAP3K2	ENSG00000169967

G2/M	
Symbol	Ensembl
IQGAP3	ENSG00000183856
TRAIP	ENSG00000183763
CDC25C	ENSG00000158402
NEIL3	ENSG00000109674
PIF1	ENSG00000140451
KIFC1	ENSG00000237649
HJURP	ENSG00000123485
NCAPH	ENSG00000121152
KIF23	ENSG00000137807
SKA3	ENSG00000165480
KIAA1524	ENSG00000163507
NDC80	ENSG00000080986
CCNF	ENSG00000162063
CDCA8	ENSG00000134690
PSRC1	ENSG00000134222
FANCD2	ENSG00000144554
ESPL1	ENSG00000135476
CDR2	ENSG00000140743
AURKB	ENSG00000178999
BORA	ENSG00000136122
LMNB1	ENSG00000113368
TRIM59	ENSG00000213186
CHEK2	ENSG00000183765
MND1	ENSG00000121211
CDCA2	ENSG00000184661
CKAP2L	ENSG00000169607
STIL	ENSG00000123473
POLQ	ENSG00000051341
MELK	ENSG00000165304
CENPL	ENSG00000120334
LIX1L	ENSG00000152022
KIF11	ENSG00000138160
C14orf80	ENSG00000185347
UBE2C	ENSG00000175063
NCAPD3	ENSG00000151503
HAUS8	ENSG00000131351
FAM83D	ENSG00000101447
CDK1	ENSG00000170312
MAD2L1	ENSG00000164109
GABPB1	ENSG00000104064
SAP30	ENSG00000164105
CFD	ENSG00000197766
TTF2	ENSG00000116830
MID1	ENSG00000101871
GAS1	ENSG00000180447
TUBA1A	ENSG00000167552
ZNF587	ENSG00000198466
TUBD1	ENSG00000108423
FAN1	ENSG00000198690
CDKN2C	ENSG00000123080
TUBB2A	ENSG00000137267
TNPO2	ENSG00000105576
ZNHIT2	ENSG00000174276
TRMT2A	ENSG00000099899
PKNOX1	ENSG00000160199
ENTPD5	ENSG00000187097
KDM4A	ENSG00000066135
STK17B	ENSG00000081320
KLF6	ENSG00000067082
KATNA1	ENSG00000186625
H2AFX	ENSG00000188486
BRD8	ENSG00000112983
RCCD1	ENSG00000166965
CDKN1B	ENSG00000111276
UACA	ENSG00000137831
KCTD9	ENSG00000104756
ATL2	ENSG00000119787
KPNA2	ENSG00000182481
HRSP12	ENSG00000132541
VTA1	ENSG00000009844
HMGB2	ENSG00000164104
C2orf69	ENSG00000178074
FADD	ENSG00000168040
HIPK2	ENSG00000064393
KIF22	ENSG00000079616
MGAT2	ENSG00000168282
NR3C1	ENSG00000113580
DHX8	ENSG00000067596
NMB	ENSG00000197696
TFAP2A	ENSG00000137203
HINT3	ENSG00000111911
CDC16	ENSG00000130177
NUMA1	ENSG00000137497
ARMC1	ENSG00000104442
STAT1	ENSG00000115415
CCDC107	ENSG00000159884
TMPO	ENSG00000120802

M	
Symbol	Ensembl
CKS1B	ENSG00000268942
DEPDC1B	ENSG00000035499
SHCBP1	ENSG00000171241
FAM64A	ENSG00000129195
FYN	ENSG00000010810
KIF2C	ENSG00000142945
SPAG5	ENSG00000076382
CIT	ENSG00000122966
CENPA	ENSG00000115163
DIAPH3	ENSG00000139734
CADM1	ENSG00000182985
KIF14	ENSG00000118193
PLK1	ENSG00000166851
MDC1	ENSG00000137337
DEPDC1	ENSG00000024526
BUB1	ENSG00000169679
DLGAP5	ENSG00000126787
NUF2	ENSG00000143228
CEP55	ENSG00000138180
GTSE1	ENSG00000075218
HMMR	ENSG00000072571
FOXM1	ENSG00000111206
E2F5	ENSG00000133740
PRR11	ENSG00000068489
NEK2	ENSG00000117650
TACC3	ENSG00000013810
CENPE	ENSG00000138778
CCNB2	ENSG00000157456
CDC20	ENSG00000117399
BIRC5	ENSG00000089685
CCDC88A	ENSG00000115355
POC1A	ENSG00000164087
MKI67	ENSG00000148773
MKI67	ENSG00000148773
HSPA13	ENSG00000155304
CDC25B	ENSG00000101224
TPX2	ENSG00000088325
AURKA	ENSG00000087586
ANKRD40	ENSG00000154945
CENPF	ENSG00000117724
CNTROB	ENSG00000170037
NCAPD2	ENSG00000010292
SGOL2	ENSG00000163535
SRF	ENSG00000112658
DZIP3	ENSG00000198919
ECT2	ENSG00000114346
ORAOV1	ENSG00000149716
NUP35	ENSG00000163002
PTPN9	ENSG00000169410
HS2ST1	ENSG00000153936
RCAN1	ENSG00000159200
SS18	ENSG00000141380
HCFC1	ENSG00000172534
NUP98	ENSG00000110713
POM121	ENSG00000196313
TOMM34	ENSG00000025772
CKAP5	ENSG00000175216
GRK6	ENSG00000198055
SEPHS1	ENSG00000086475
QRICH1	ENSG00000198218
AHI1	ENSG00000135541
CNOT10	ENSG00000182973
KLF9	ENSG00000119138
SETD8	ENSG00000183955
ATF7IP	ENSG00000171681
RAD51C	ENSG00000108384
CDC42EP1	ENSG00000128283
HPS4	ENSG00000100099
GOT1	ENSG00000120053
MZT1	ENSG00000204899
RRP1	ENSG00000160214
AKIRIN2	ENSG00000135334
CDC27	ENSG00000004897
SMARCD1	ENSG00000066117
BIRC2	ENSG00000110330

M/G1	
Symbol	Ensembl
TROAP	ENSG00000135451
CDKN3	ENSG00000100526
PRC1	ENSG00000198901
HSD17B11	ENSG00000198189
BTBD3	ENSG00000132640
SLC39A10	ENSG00000196950
GTF3C4	ENSG00000125484
WWC1	ENSG00000113645
ELP3	ENSG00000134014
FOXK2	ENSG00000141568
OPN3	ENSG00000054277
KIAA0586	ENSG00000100578
ANTXR1	ENSG00000169604
CEP70	ENSG00000114107
HMGCR	ENSG00000113161
TULP4	ENSG00000130338
ZNF281	ENSG00000162702
CDK7	ENSG00000134058
LYAR	ENSG00000145220
PPP6R3	ENSG00000110075
DCP1A	ENSG00000162290
FAM189B	ENSG00000160767
AGPAT3	ENSG00000160216
PSEN1	ENSG00000080815
NUP37	ENSG00000075188
MSL1	ENSG00000188895
AGFG1	ENSG00000173744
SNUPN	ENSG00000169371
STAG1	ENSG00000118007
LRIF1	ENSG00000121931
PAK1IP1	ENSG00000111845
NCOA3	ENSG00000124151
PTTG1	ENSG00000164611
CTR9	ENSG00000198730
DKC1	ENSG00000130826
FOPNL	ENSG00000133393
VCL	ENSG00000035403
MRPS2	ENSG00000122140
WIPF2	ENSG00000171475

Supplementary Table 1: Gene sets used to assess enrichment of cell cycle phases related transcripts in individual cells analyzed by scRNA-seq.

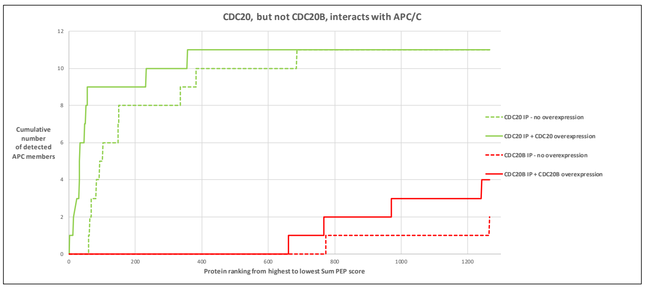

Protein ranking from highest to lowest Sum PEP score	Cumulative number of detected APC/C members			
	CDC20 IP - no overexpression	CDC20 IP + CDC20 overexpression	CDC20B IP - no overexpression	CDC20B IP + CDC20B overexpression
1	0	0	0	0
2	0	1	0	0
6	0	1	0	0
7	0	1	0	0
12	0	1	0	0
13	0	2	0	0
24	0	3	0	0
27	0	3	0	0
28	0	3	0	0
30	0	3	0	0
31	0	4	0	0
32	0	5	0	0
33	0	6	0	0
46	0	6	0	0
47	0	7	0	0
50	0	7	0	0
51	0	8	0	0
55	0	8	0	0
56	0	9	0	0
59	0	9	0	0
60	1	9	0	0
62	1	9	0	0
63	2	9	0	0
67	2	9	0	0
68	3	9	0	0
81	3	9	0	0
82	4	9	0	0
91	4	9	0	0
92	5	9	0	0
101	5	9	0	0
102	6	9	0	0
147	6	9	0	0
148	7	9	0	0
149	7	9	0	0
150	8	9	0	0
231	8	9	0	0
232	8	10	0	0
335	8	10	0	0
336	9	10	0	0
356	9	10	0	0
357	9	11	0	0
382	9	11	0	0
383	10	11	0	0
660	10	11	0	0
661	10	11	0	1
685	10	11	0	1
686	11	11	0	1
767	11	11	0	1
768	11	11	0	2
773	11	11	0	2
774	11	11	1	2
970	11	11	1	2
971	11	11	1	3
1241	11	11	1	3
1242	11	11	1	4
1265	11	11	1	4
1266	11	11	2	4

IP CDC20 - no overexpression

Protein FDR Confidence	Accession	Symbol	Description	Sum PEP Scor	Coverage	# Peptides	# PSMs	# Unique Peptides	MW [kDa]	emPAI	Score Sequest HT	global rank	APC rank
High	Q9UJX2	CDC23	Cell division c	109,78	47,74	27	48	27	68,79	6,80	88,85	60	1,00
**High**	**Q12834**	**CDC20**	**Cell division c**	**104,51**	**49,70**	**16**	**56**	**16**	**54,69**	**10,94**	**158,30**	63	2,00
High	Q9H1A4	ANAPC1	Anaphase-pro	101,92	23,66	32	42	32	216,36	1,30	36,79	68	3,00
High	Q9UJX4	ANAPC5	Anaphase-pro	91,89	42,38	23	50	23	85,02	3,19	83,00	82	4,00
High	Q9UJX3;A0A024RBJ3	ANAPC7	Anaphase-pro	85,18	46,58	21	38	21	66,81	6,13	61,05	92	5,00
High	A0A024RDZ2;Q13042	CDC16	CDC16 cell div	79,32	43,55	21	31	21	71,61	4,37	48,14	102	6,00
High	P30260	CDC27	Cell division c	63,63	26,33	16	31	1	91,81	2,00	41,46	148	7,00
High	G5EA36	CDC27	Cell division c	63,28	26,25	16	31	1	91,68	2,00	41,49	150	8,00
High	Q9UJX6	ANAPC2	Anaphase-pro	37,09	22,75	13	15	13	93,77	0,93	15,24	336	9,00
High	Q9UJX5	ANAPC4	Anaphase-pro	31,69	16,34	13	13	13	92,06	0,89	7,67	383	10,00
High	Q9UM13	ANAPC10	Anaphase-pro	16,01	31,89	5	9	5	21,24	1,85	11,56	686	11,00

IP CDC20 + CDC20 overexpression

Protein FDR Confidence	Accession	Symbol	Description	Sum PEP Scor	Coverage	# Peptides	# PSMs	# Unique Peptides	MW [kDa]	emPAI	Score Sequest HT	global rank	APC rank
High	Q9H1A4	ANAPC1	Anaphase-pro	710,46	59,57	81	319	81	216,36	28,94	640,59	2	1
High	Q9UJX4	ANAPC5	Anaphase-pro	463,89	66,23	50	288	46	85,02	142,07	635,28	13	2
High	Q9UJX5	ANAPC4	Anaphase-pro	371,59	59,53	43	198	43	92,06	34,74	384,67	24	3
**High**	**Q12834**	**CDC20**	**Cell division c**	**332,93**	**69,54**	**28**	**791**	**28**	**54,69**	**202,09**	**1712,61**	28	3
High	P30260	CDC27	Cell division c	306,64	60,56	34	215	1	91,81	20,92	435,97	31	4
High	G5EA36	CDC27	Cell division c	306,44	60,51	34	212	1	91,68	20,92	426,79	32	5
High	Q9UJX6	ANAPC2	Anaphase-pro	301,10	54,50	45	165	45	93,77	27,12	297,57	33	6
High	A0A024RDZ2;Q13042	CDC16	CDC16 cell div	253,91	55,00	29	108	29	71,61	22,90	226,42	47	7
High	Q9UJX2	CDC23	Cell division c	244,49	54,77	33	121	33	68,79	27,80	262,31	51	8
High	Q9UJX3;A0A024RBJ3	ANAPC7	Anaphase-pro	236,24	64,61	32	117	32	66,81	37,75	239,65	56	9
High	Q9UM13	ANAPC10	Anaphase-pro	86,65	76,22	11	43	11	21,24	27,48	98,76	232	10
High	I3L3H6	CDC27	Cell division c	63,60	63,09	7	32	1	25,91	5,81	75,14	357	11

IP CDC20 B- no overexpression

Protein FDR Confidence	Accession	Symbol	Description	Sum PEP Scor	Coverage	# Peptides	# PSMs	# Unique Peptides	MW [kDa]	emPAI	Score Sequest HT	global rank	APC rank
High	Q9UJX5	ANAPC4	Anaphase-pro	7,09	3,47	1	2	1	92,06	0,05	2,13	774	1
High	A0A024RDZ2;Q13042	CDC16	CDC16 cell div	2,31	4,68	1	1	1	71,61	0,06	0,00	1266	2

IP CDC20 B + CDC20B overexpression

Protein FDR Confidence	Accession	Symbol	Description	Sum PEP Scor	Coverage	# Peptides	# PSMs	# Unique Peptides	MW [kDa]	emPAI	Score Sequest HT	global rank	APC rank
**High**	**Q86Y33**	**CDC20B**	**Cell division c**	**215,12**	**60,89**	**26**	**271**	**26**	**57,30**	**41,99**	**546,93**	7	0
High	Q9UJX2	CDC23	Cell division c	11,98	12,90	5	5	5	68,79	0,37	0,00	661	1
High	A0A024RDZ2;Q13042	CDC16	CDC16 cell div	9,77	5,65	3	3	3	71,61	0,21	3,53	768	2
High	P30260	CDC27	Cell division c	6,89	3,64	3	3	3	91,81	0,17	3,63	971	3
High	Q9UJX3;A0A024RBJ3	ANAPC7	Anaphase-pro	4,21	6,18	2	2	2	66,81	0,15	0,00	1242	4

Supplementary Table 2: CDC20, but not CDC20B, interacts with APC/C.
